# Femtosecond‐Laser‐Induced Physical Unclonable Random Maze Structure for Storage‐Free Encryption

**DOI:** 10.1002/advs.75973

**Published:** 2026-06-11

**Authors:** Shiru Jiang, Hongliang Li, Shengjie Ma, Sang‐Shin Lee, Lei Wang, Mengyun Hu, Heping Zeng

**Affiliations:** ^1^ State Key Laboratory of Precision Spectroscopy Hainan Institute East China Normal University Shanghai China; ^2^ School of Information Science and Technology Qingdao University of Science and Technology Qingdao China; ^3^ Chongqing Key Laboratory of Precision Optics Chongqing Institute of East China Normal University Chongqing China; ^4^ Key Laboratory of Multifunctional Nanomaterials and Smart Systems Suzhou Institute of Nano‐Tech and Nano‐Bionics Chinese Academy of Sciences Suzhou China; ^5^ Department of Electronic Engineering Kwangwoon University Seoul South Korea; ^6^ College of Mathematics and Physics Qingdao University of Science and Technology Qingdao China; ^7^ Institute of Precision Optical Equipment for Marine Surveys (POEMS) Yazhou Bay Science and Technology City Sanya Hainan China

**Keywords:** femtosecond laser, gold random maze structure, marangoni convection, on‐site generated key, physical unclonable functions

## Abstract

Physical unclonable functions (PUFs) serve as physical cryptographic primitives that provide hardware‐level information protection. However, as PUFs shrink to smaller and more complex dimensions, they face increasing challenges in response accessibility and storage overhead. Here, we present a multi‐level, easily accessible PUFs based on gold random maze structures that enable information encryption without storing responses or keys. The mazes are formed through femtosecond‐laser‐induced localized plasmonic effects and Marangoni convection on gold near‐percolation films. The random morphologies support independent optical and electrical PUFs based on Au mazes. The microscale interconnects endowed with nanoscale structural characteristics allow on‐site acquisition of PUF responses and real‐time key generation during encryption with high security. We further demonstrate a high‐security and storage‐free encryption process on an integrated circuit platform, highlighting a path to shift from long‐term key storage models to next‐generation, on‐site security architectures.

## Graphical abstract

Femtosecond‐laser‐induced gold random maze structures serve as multimodal physical unclonable functions for storage‐free encryption. Their stochastic optical, electrical, and Raman responses are generated by plasmon‐assisted Marangoni formation and converted into AES‐compatible keys without permanent secret‐key storage, offering a portable route toward secure on‐site authentication and hardware encryption.

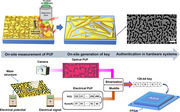

## 1 Introduction

With the explosive development of embedded artificial intelligence (AI) and the Internet of Things (IoT), there is a growing demand for powerful and invulnerable security systems to protect personal information [1, 2]. Current mainstream security strategies primarily rely on complex software algorithms to generate random numbers (i.e., cryptographic keys), which are then stored in the internal non‐volatile memory of devices for authentication [3, 4]. However, the advent of machine learning and quantum computing has severely challenged the reliability of such approaches: so‐called “complex algorithms” are increasingly vulnerable to cryptanalysis, and the very act of storing keys presents serious security risks [5, 6]. For example, the Advanced Encryption Standard (AES), a cornerstone of modern cryptography, requires its private key to be stored in non‐volatile memory for encryption and decryption [7]. Once compromised, the leakage of this stored key would completely expose the private information of users [8]. In parallel, to enable efficient computation, AI devices typically store trained neural network parameters (i.e., weights) locally in computing‐in‐memory modules [8]. This practice introduces critical security breaches: weight data on the chip can be stolen by simply reading the memory data through the legal Input/Output ports, then the whole neural network can be copied, and the intellectual property of designers is at risk [1, 9, 10].

For better security, physical unclonable functions (PUFs) have emerged as a promising hardware‐based security paradigm [11, 12, 13, 14, 15]. By exploiting the inherent randomness of microscopic physical structures, PUFs enable device authentication and cryptographic key generation at the hardware level, offering a more secure alternative to conventional software‐based methods [16, 17, 18]. However, some conventional PUF and anti‐counterfeiting schemes may still face practical security challenges under advanced characterization, imaging‐assisted reverse engineering, physical replication, or machine‐learning‐based modeling attacks, especially when the physical features are relatively simple or repeatedly accessible [12, 19]. It was not until the advent of nanoscale PUFs that hardware‐based authentication achieved a substantial leap in security, redefining the foundation of modern anti‐counterfeiting technologies. Nevertheless, the drastic reduction in physical dimensions inevitably undermines the usability and user‐friendliness in a real scene. As a result, most PUF technologies that lack market compatibility remain confined to academic and research laboratories, rather than being widely deployed in practical, real‐world applications [13, 15]. As is well known, due to the inherent complexity, the unique physical characteristic of each PUF necessitates intricate challenge‐response measurements during production. This process typically relies on high‐precision and costly instruments, such as scanning electron microscopes (SEM) and professional spectroscopic devices, making it time‐consuming, expensive, and demanding in terms of data storage—particularly when handling large volumes of high‐resolution image data. This not only complicates the authentication process but also limits the practical implementation in resource‐constrained IoT devices [12, 19, 20, 21, 22]. Furthermore, complicated and multi‐step fabrication requirements, poor environmental stability, and limited compatibility with cryptographic modules notably hinder the pace of marketization of nanoscale PUFs, too [11, 13]. The above challenges, especially the irreconcilable contradiction between the security and practicality of PUFs, highlight the necessity of developing the next‐generation robust and storage‐free (both of PUF responses and keys) anti‐counterfeiting technologies.

In this work, based on femtosecond‐laser (fs‐laser)‐induced Au random maze structures (RMSs), a PUF architecture simultaneously enabling high security and usability is realized, which can provide storage‐free and multi‐dimensional hardware system protection with on‐site generated keys. Irradiation of fs‐laser on the Au near‐percolation film surface excites the strong localized surface plasmon resonance (LSPR) and local heat confinement, which triggers the Marangoni convection instability and ultimately results in RMSs. The RMS is composed of random fractal networks in unpredictable height that are the origin of nonuniform electrical conductivity, and the random Raman spectra can be detected by sandwiching a graphene layer between the Au and substrate, before fs‐laser irradiation. Thus, a single Au RMS can generate multiple, independently optical, electrical, and Raman PUFs, corresponding to the fractal morphology, electrical resistance, and Raman signatures, respectively. The challenge–response pairs of optical and electrical PUFs can be conveniently executed by end users, enabling secure, on‐site, and on‐demand key generation without data storage. This approach effectively eliminates the cumbersome characterization and massive data storage required in conventional PUFs, thereby avoiding data leakage and excessive hardware overhead. Furthermore, the Au RMSs are fabricated on SiO_2_/Si substrates sharing the same hierarchical structure as metal–oxide–semiconductor field‐effect transistors (MOSFETs), ensuring compatibility with integrated hardware platforms. Finally, we demonstrate practical on‐site key generation using a smartphone and a multimeter, and validate its application in an FPGA‐based AES encryption system, showcasing the potential of Au RMSs for next‐generation storage‐free security architectures.

## 2 Results and Discussion

### 2.1 RMS PUFs Enabled Storage‐Free Encryption

**FIGURE 1:**
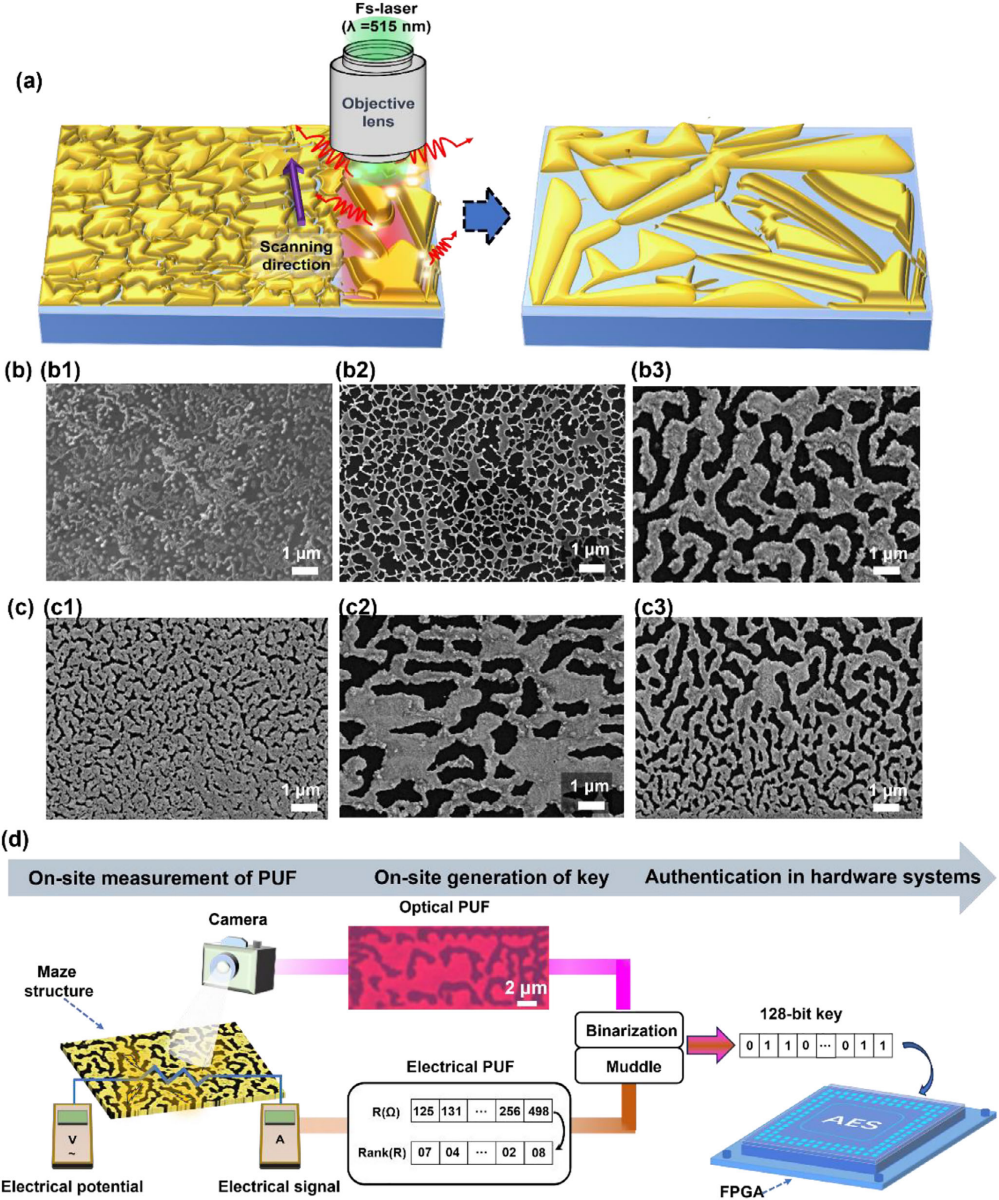
Generation of on‐site RMS PUFs and key for storage‐free encryption. (a) Schematic of the fabrication of Au RMS by LSPR‐FLDW. The violet arrow represents the direction of sample scanning. (b) SEM image of RMS fabricated on Au percolation films with the film thickness (*t*) of (b1) 5, (b2) 10, and (b3) 20 nm. (c) SEM images of RMSs on 20‐nm‐thick Au percolation film with different parameters of LSPR‐FLDW, where pulse energy and NA of objective lens of (c1–c3) were 8.1 nJ & 0.6, 10 nJ & 0.3, and 8.1–8.9 nJ & 0.6, respectively. (d) Conceptual schematic of generating the storage‐free and on‐site PUF key and its application in the hardware encryption system. The optical PUF in (d) is fabricated with a 0.3‐NA lens and FPGA is Field Programmable Gate Array.

The generation of the gold RMS via a LSPR‐assisted fs‐laser direct writing (LSPR‐FLDW) is described schematically in Figure 1a. On a SiO_2_/Si substrate, the 20‐nm‐thick Au near‐percolation film comprising many isolated nanoislands was deposited, which provides sufficient hotspots from the LSPR excited by a 515‐nm fs‐laser beam with a repetition rate of 500 kHz. These hotspots lead to a strongly localized heat distribution, then Marangoni convection [23] dominates the flow of molten gold and ultimately forms Au RMSs, as shown in Figure 1b3,c. The random distribution of hotspots and the uncontrolled Marangoni convection are the sources of the unpredictability of the fractal structure and electrical conductivity within the Au RMS. The nanoscale randomness in fractal structures, especially the uneven height along the maze edges, endows the Au RMS with security comparable to that of nanoscale PUFs. The distinguishing characteristics of the RMS from normal dewetting structures in Au thin films are its excellent connectivity and the wide tunable range of its feature size. As shown in Figure 1b and Figure S1, the average feature size of RMS changes from 310 nm to 1.3 µm when the thickness (*t*) of Au thin film increased from 5 to 20 nm. In addition, the pulse energy of fs‐laser and numerical aperture (NA) of objective lens (Figure 1c and Figures S2 and S3) can effectively tune the feature size of the RMS from the nanoscale to the microscale. When the fs‐laser pulse energy and NA of the objective lens were carefully selected, micron‐scale RMSs can be readily obtained on a 20 nm‐thick Au film, as shown in Figure 1b3,c2, c3, which allows the RMS to be characterized as an optical PUF in an easy way, for example, a smartphone connected with a portable mini‐microscope, ensuring user‐friendly operation. The obtained optical and electrical (i.e., resistance) PUFs are processed to generate unique and highly secure on‐site keys through binarizing, hashing, and muddling algorithms. Consequently, the entire process, including the acquisition, processing, and final key generation of optical and electrical PUFs, can be completed by the end‐users in real time and on‐site. This approach not only overcomes the drawbacks of traditional pre‐stored data but also eliminates the common trade‐off between security and operability in conventional PUF systems, while addressing the resource constraints of IoT devices [11]. Figure 1d illustrates conceptually the above process, where the final key is formed via muddling optical and electrical PUFs, and the FPGA (Field Programmable Gate Array)‐based AES system is used to represent resource‐constrained IoT devices.

### 2.2 Formation Mechanism of Au RMS

**FIGURE 2:**
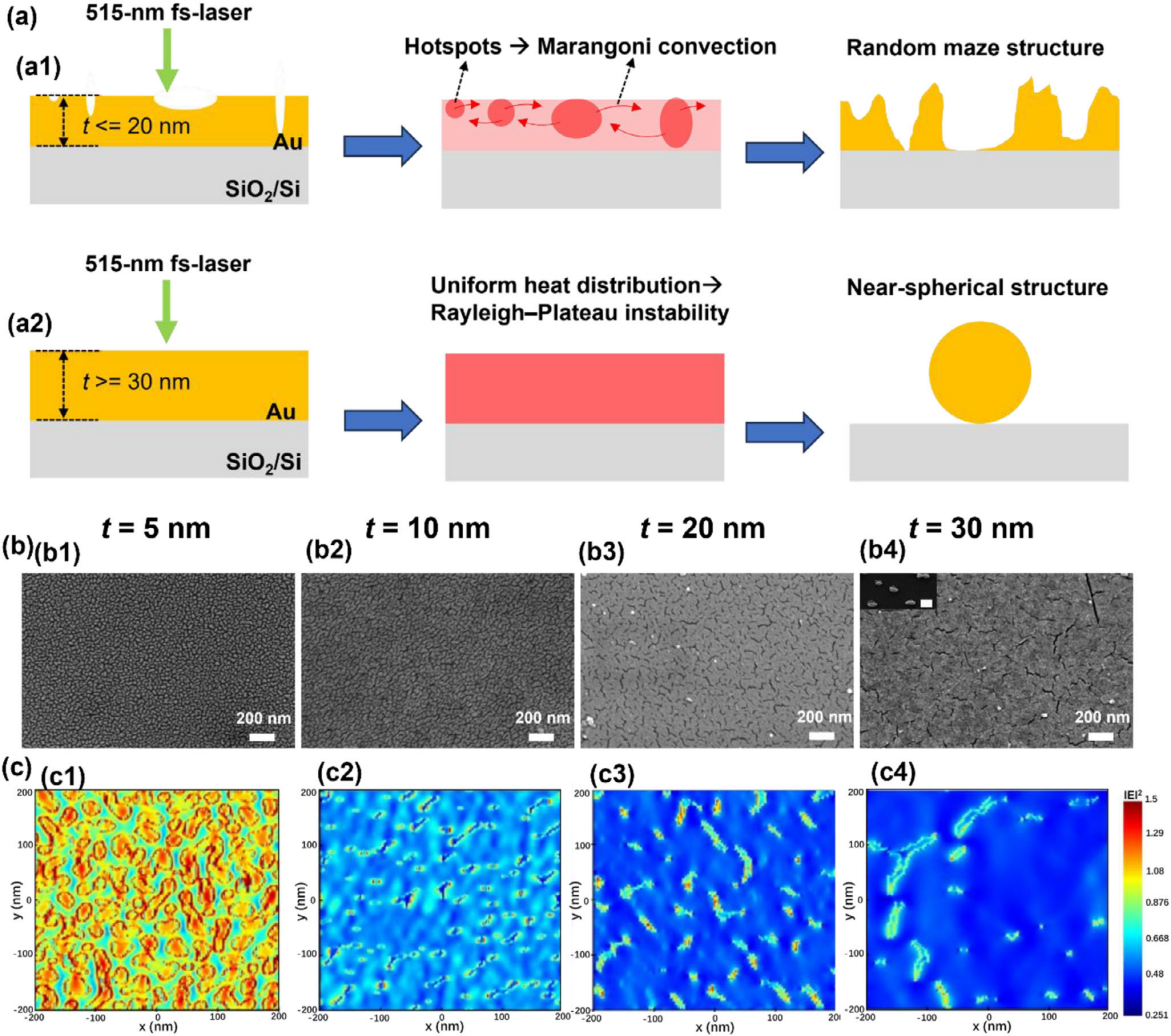
Operation mechanism of Au RMSs. (a) Schematic of the (a1) RMS and (a2) near‐spherical structure formation process in terms of *t* ≤ 20 nm and *t* ≥ 30 nm, respectively. (b) SEM images of Au percolation films before LSPR‐FLDW with *t* of 5, 10, 20, and 30 nm. Insert in (b4) is the near‐spherical structure after fs‐laser treatment for a 30‐nm‐thick Au film and the scale bar is 2 µm. (c) Simulated electric (E) field distributions of Au surfaces reconstructed corresponding to (b) in response to a plane wave with a wavelength of 515 nm.

The formation of RMS is not driven by a conventional thermally driven metal dewetting process, but rather a synergistic interplay of multiple nonequilibrium physical mechanisms. In particular, the emergence of RMS is jointly governed by plasmon‐enhanced absorption, Marangoni convection, complex molten metal flow, and ultrafast nonequilibrium thermal dynamics induced by femtosecond laser irradiation, revealing that the emergence of RMS is predominantly governed by plasma effect and Marangoni convection [23]. As shown in Figure 2a1, Au near‐percolation films (*t* < 30 nm) with numerous hotspots are more preferable for forming highly localized thermal distributions, which is mainly attributed to the highly concentrated heat sources at the hotspots and the remarkable spatial geometric gradients of the nanoislands. These hotspots created dense regions of elevated energy deposition and heterogeneous melting points, then steep temperature and surface tension gradients. Subsequently, Marangoni convection began to dominate material migration within the molten layer, which led to irregular flow and bifurcation in the liquid Au via combining with perturbations such as thermocapillary and thermal stress‐driven flows [23]. At the same time, because the melting sites evolving from hotspots inherited the characteristics of high density and the shortest distance, interconnection between adjacent melting sites occurred and gradually developed across the entire surface. The ultrafast kinetics from fs‐laser irradiation induced rapid resolidification, which effectively froze fractal structures in the non‐equilibrium state before the molten Au shrinking into isolated spheres [23], and the proposed RMS formed.

To support the proposed formation mechanism, the LSPR behavior of Au near‐percolation films with different thicknesses *t* was investigated. The SEM images in Figure 2b display that the number of nanoislands decreases with the increased *t*, which leads to a reduction in the number (Figure 2b) and density (Figure S4a) of hotspots after exciting LSPR on Au surfaces by a 515‐nm fs‐laser. Accordingly, the shortest distance between hotspots exhibits a positive correlation with *t*, as shown in Figure S4b. Depending on finite element simulations based on the two‐temperature model [24], the heat source originating from plasmonic hotspot absorption exhibits much stronger spatial localization in both electron and lattice temperature, compared with the case of uniform bulk absorption (See Figure S5c,d and Note S1 for details). This explains that the thinner Au films with more nanoislands will lead to more complex structures and smaller feature sizes of the RMS as shown in Figure 1b, because more and stronger Marangoni convections independently form due to larger temperature gradients. Therefore, *t* serves as an effective parameter for tuning the RMS feature size from the nanoscale to the microscale. Note that there are only some nanoparticles in the shape of hills instead of any fractal structure when *t* = 30 nm (Figure 2b4), even with elaborately optimizing various parameters of LSPR‐FLDW. This is a result of the fact that the 30‐nm‐thick Au film tends to follow the uniform bulk absorption and thermal diffusion (Figure S5a,c) due to a weaker plasmonic effect (Figure 2c4 and Figure S4). Figure 2a2 schematically illustrates this process, where the Rayleigh–Plateau instability, instead of Marangoni convection, dominates the flow of liquid gold. Due to the same reason, the 20‐nm‐thick Au near‐percolation film under the irradiation of a 1030‐nm fs‐laser failed to grow the RMS as displayed in Figure S6, which can be interpreted by the feeble plasmon effects of Au nanoislands in the near‐infrared compared with the visible range [25].

The RMS formation reported here follows a distinct nonequilibrium patterning pathway, fundamentally different from conventional laser‐induced metal dewetting. In typical dewetting, molten metal evolves under quasi‐uniform thermal fields and is driven by surface‐energy minimization, directly yielding isolated spherical particles without any intermediate networked morphology [11]. By contrast, RMS originates from plasmonic‐hotspot‐dominated, highly nonuniform energy deposition, which creates densely coupled melting sites and drives lateral interconnection and branching via dominant Marangoni convection rather than isotropic contraction. The ultrafast melting–resolidification dynamics enabled by femtosecond laser irradiation further suppress the transition toward thermodynamically stable spheres, stabilizing a maze‐like fractal network as an intrinsic outcome of this process. Only when this balance is intentionally disrupted, for example by excessively increasing the pulse energy, does the RMS gradually degenerate into near‐spherical particles (Figure S2), whereas reducing the film thickness t instead enhances hotspot density and structural complexity. Together with the additional control provided by the laser spot size and scanning overlap ratio (Figures S3, S7, and S8), these results demonstrate that RMS is not a truncated form of dewetting, but a distinct plasmon‐assisted, femtosecond‐laser‐enabled regime.

### 2.3 Performance of the PUFs

**FIGURE 3:**
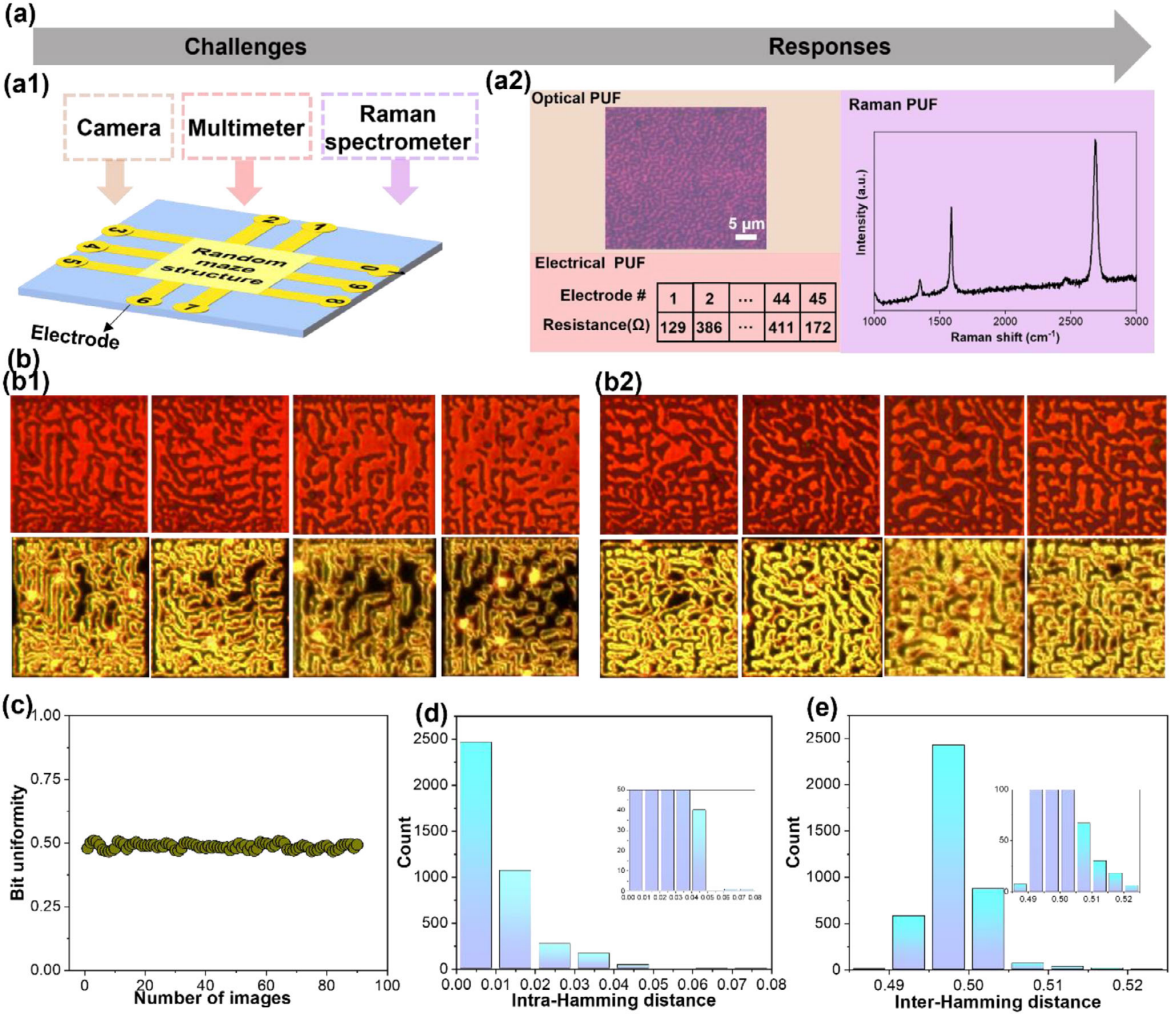
Performance tests of PUFs based on Au RMS. (a) Multidimensional challenge‐response authentication process based on Au RMS. (b) Brightfield (the above) and darkfield (the below) optical micrographs of Au RMSs representing optical and electrical PUFs, respectively, fabricated by LSPR‐FLDW with pulse energies of (b1)10.3 and (b2) 10.9 nJ and 0.3‐NA objective lens on a 20‐nm‐thick Au near‐percolation film. The size of all optical micrographs in (b) is 30 × 30 µm^2^. (c) Bit of uniformity from the occurrence probability of “1” in binary bits extracted from 90 optical PUF images. (d) Intra‐HD supported by 90 optical PUF images by repeatedly measuring 90 times for a single RMS. (e) Inter‐HD based on 90 optical PUF images obtained from different RMSs. A total of 45 resistance values of electrical PUF in (a2) were measured based on the 10 electrode arrangements (C(10, 2) = 45) illustrated in (a1).

The Au RMS can respond to multiple challenges for creating optical, electrical, and Raman PUFs via independent physical mechanisms—optical images, electrical resistance, and Raman spectrum based on fractal network, conductivity, and additional single‐layer graphene, respectively—empowering independent anti‐counterfeiting methods as shown in Figure 3a. The optical and electrical PUFs are enabled by the unpredictability of Au RMS, originating from stochastically distributed hotspots and inscrutable Marangoni flow during its formation. To verify the intrinsic randomness and unpredictability, eight RMS are fabricated in two sets of four, as displayed in Figure 3b, where the brightfield and darkfield micrographs are used to represent the optical and electrical PUFs featuring disordered connections, respectively. Identical parameters of LSPR‐FLDW were applied within each set, but all RMS exhibited distinct patterns. When using the Au near‐percolation film with *t* = 20 nm, the feature size of RMSs enlarged to the micrometer range (Figure 1c), which facilitates the optical and electrical PUFs to be conveniently detected via a portable camera and multimeter. This is important for realizing the practical and wide application of the proposed anti‐counterfeiting architecture [26]. The random Raman spectrum is also an effective anti‐counterfeiting scheme [19], which was used as the third‐dimension anti‐counterfeiting in this work, characterizing the structural damage information of single‐layer graphene (Figure 3a2). Attributed to the efficient capability of structural replication from thin Au film to graphene [27, 28], it can be inferred that similar RMSs can be created in the single‐layer graphene, which was deposited between the Au and SiO_2_/Si substrate as shown in Figure S9. Thus, the Raman spectra also possess unpredictability even under the same fabrication conditions (Figure S10).

Benefiting from the multilevel and multidimensional anti‐counterfeiting capabilities of the Au RMS, users can flexibly select an authentication method tailored to specific application requirements. When high security is the top priority, thinner RMS (e.g., the case of *t* = 5 nm in Figure 1b1) with additional single‐layer graphene can be employed, leveraging nanoscale features and the inherent difficulty of characterization. Conversely, if the ease of use and cost‐effectiveness dominate the requirements, a 20‐nm‐thick RMS (Figure 1c) will provide an optimal choice. In this low‐cost optical/electrical mode, the feature size of RMS increases to the microscale, which can be conveniently read out by using portable microscopes or even smartphones equipped with auxiliary devices (Figure S11), where the fs‐laser is required only for tag fabrication, whereas routine authentication and on‐site key generation rely on portable optical imaging and electrical resistance measurement. The corresponding fabrication/readout cost estimation is provided in Note S2. Therefore, the optical and electrical PUFs enable a user‐friendly and on‐demand encryption mode while maintaining high security through the combination of the two PUFs.

To quantitatively evaluate the performance of the anti‐counterfeiting scheme based on Au RMS, three typical metrics, including bit uniformity, interchip Hamming distance (inter‐HD), and intrachip Hamming distance (intra‐HD), were calculated to assess the randomness, uniqueness, and reliability of the PUFs, respectively. Here, taking the optical PUF based on RMSs as a representative channel for full quantitative response/key evaluation, each optical PUF generated a 256‐bit binary string after binarization and SHA‐256 hashing (Figure S12). Bit uniformity represents the ratio of the number of ‘1’ to the total number of bits in the response binary string, whose ideal value is 0.5 for a perfectly random distribution. The bit uniformity in Figure 3c exhibits a nearly linear trend close to 0.5 with an average of 0.487, indicating randomness and unpredictability in optical PUFs. The intra‐HD is used to evaluate the variation within the same PUFs. After measuring the same optical PUF 90 times, the average intra‐HD value was 0.0094, near to the ideal value of 0, verifying minimal discrepancy between the same PUFs. The non‐zero values in Figure 3d can be explained by CCD noise, small vibrations in the optical system, and fluctuations in the light source [3]. In contrast, the average inter‐HD in Figure 3e is 0.4981, approaching the theoretical value of 0.5, demonstrating excellent uniqueness among different PUFs (calculations can be found in Note S3). Furthermore, Shannon entropy, min‐entropy, and NIST SP 800‐22‐style tests were conducted to further evaluate their statistical randomness and worst‐case predictability. The responses exhibited an average Shannon entropy of 0.999175, an average min‐entropy of 0.960151, and passed the NIST‐style randomness tests, confirming the high statistical randomness and low predictability of the generated responses (See Figure S13, Table S4, and Note S4 for details). In addition, Lempel–Ziv (LZ) entropy [29] and the bit error rate (BER) [13] were calculated to evaluate the long‐term stability and environmental robustness of the PUFs. The relatively small variations in both LZ entropy and BER suggest that the statistical complexity, randomness, and reliability of the PUF responses can be largely maintained within the tested aging period and temperature range, indicating the promising stability of the Au RMS‐based PUFs under the present experimental conditions. (See Figure S14 and Note S5 for details).

### 2.4 On‐Site Generated PUF Key for Storage‐Free Encryption

**FIGURE 4:**
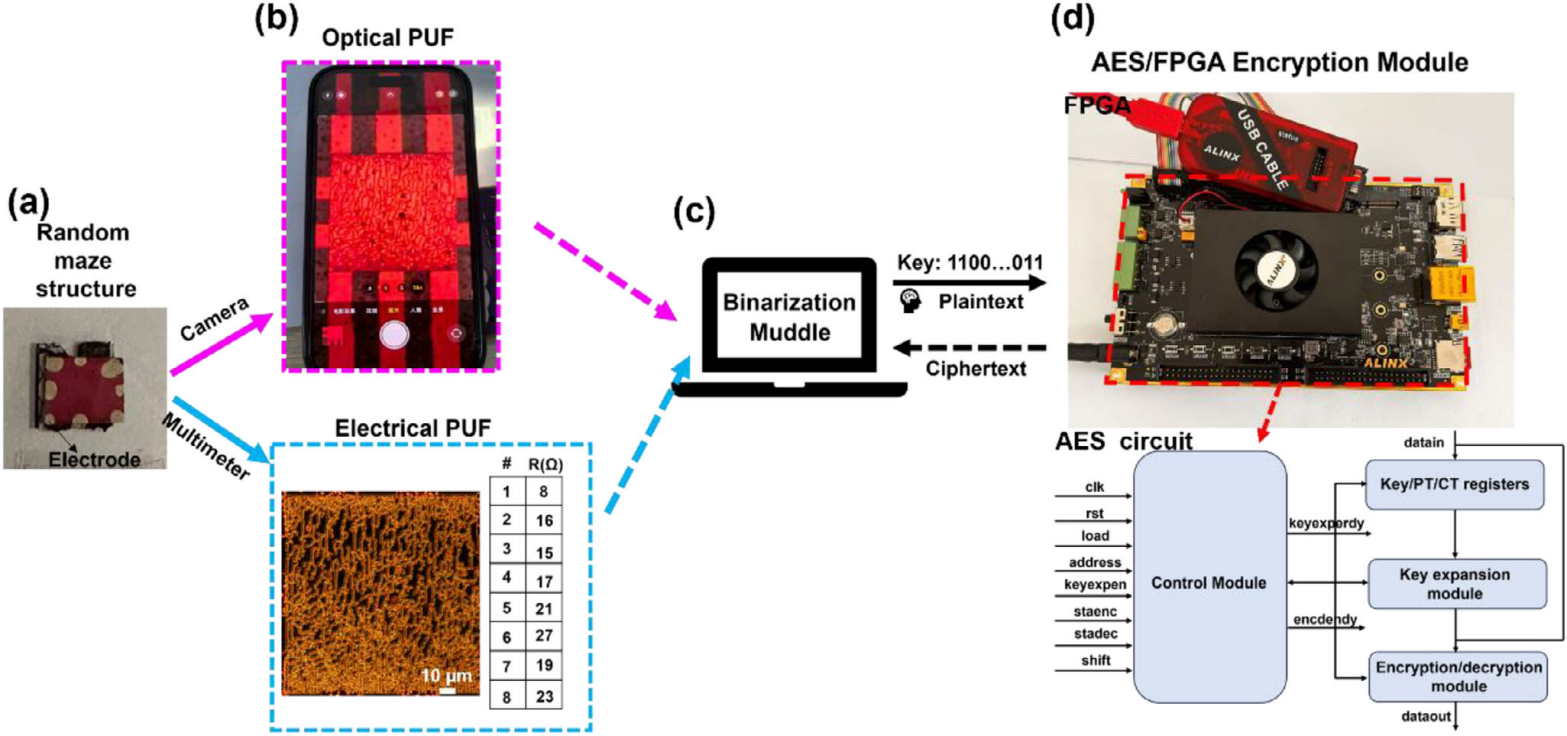
The process of using on‐site‐generated key for storage‐free AES symmetric encryption. (a) Au RMS used for on‐site generated PUF key system. (b) Optical and electrical PUFs obtained by portable devices. (c) Generation of a 128‐bit key by binarization and muddle operation. (d) AES/FPGA encryption module.

Profiting by the convenient operation to obtain PUFs on Au RMS via portable devices (Figure S11 and Figure 4b), challenge‐response pairs or keys can be generated in real‐time and on‐site each time requirements emerge, which supports the data storage‐free security and encryption systems. Therefore, forming a general‐purpose encryption framework that requires no long‐term storage of keys or sensitive data. A critical feature of this framework is its algorithm‐agnostic nature: the generated keys serve as a trusted entropy source that can be flexibly combined with mainstream symmetric and asymmetric cryptographic algorithms, supporting diverse application modes such as local data protection and secure communication. For asymmetric encryption scenarios, a storage‐free secure communication protocol based on on‐site key generation is illustrated through a system‐level workflow, with the complete interaction process and security logic presented in Figure S15 (See Note S6 for details).

To further illustrate the feasibility and engineering compatibility of the proposed storage‐free security system on practical hardware platforms, AES as a representative example of symmetric encryption was selected. AES is widely adopted in industrial and embedded systems with high amenability to hardware implementation. Using a commercial integrated‐circuit development platform (FPGA), storage‐free encryption originating from on‐site generated keys was demonstrated in Figure 4. It should be emphasized that AES is employed here not as a restrictive choice, but as a standardized and hardware‐friendly instance to illustrate the broad interoperability of the proposed framework with existing cryptographic ecosystems. In some medical devices and neural‐network systems, optical and electrical PUFs are first acquired using portable devices from an Au RMS as illustrated in Figure 4a,b. The PUFs are then processed by binarization, hashing, and muddling operations are performed to generate a 128‐bit key (Figure 4c and Figure S12). Here, the hashing and muddling operations are used as multimodal response‐fusion steps to improve bit diffusion, response uniformity, and cryptographic compatibility, rather than as artificial entropy sources. The entropy of the generated key originates from the physical randomness of the Au RMS morphology and the independently acquired optical/electrical PUF responses [13, 30]. This key is dynamically loaded into the AES module implemented on the FPGA for encrypting sensitive local information (plaintext), such as patient information or trained model weights in neural‐network systems. The encrypted information as ciphertext is written into storage units, and waits for the decryption when the next computation is required (See Table S5 and Movie S1). Notably, after each encryption or decryption operation, the generated key is immediately erased and regenerated on demand for subsequent use, ensuring that no persistent secret exists within the system. This strategy mitigates key‐leakage risks and limits the accumulation of reusable response/key data for potential machine‐learning attacks. In practical authentication scenarios, replay attacks can be further mitigated by incorporating a verifier‐provided nonce, timestamp, or session challenge into the key‐derivation process, so that previously recorded responses or keys cannot be reused in later sessions. In addition, considering the resource constraints commonly encountered in IoT scenarios, the AES hardware adopts a time‐multiplexed architecture in which encryption and decryption circuitry are shared; the corresponding circuit schematics and signal characteristics are provided in Figure S16 and Table S6. This on‐demand and ephemeral key management strategy fundamentally resolves the long‐standing trade‐off between security and practicality in conventional PUF‐based systems, while enabling seamless compatibility with different cryptographic paradigms and hardware platforms. Thus, the proposed approach highlights strong potential for practical deployment and industrial integration.

## 3 Conclusions

A multidimensional, user‐friendly, storage‐free, and on‐site key generation system based on Au RMS is demonstrated. By activating LSPR and Marangoni flow, Au RMS was formed after fs‐laser irradiating Au near‐percolation films. Benefiting from the micron‐scale feature size and excellent electrical connectivity of the Au RMS, its fractal morphology and resistance can be conveniently obtained by users through portable devices, enabling a practical encryption system with on‐site key generation. This storage‐free encryption approach can effectively eliminate the risks of key/response leakage and limited storage resources faced by conventional pre‐stored data schemes. Meanwhile, the simple operation for gaining PUFs and generating keys enables a wider range of applications and scenarios. Importantly, reducing the gold film thickness allows the RMS to reach the nanoscale, which expands the anti‐counterfeiting capacity and meets diverse application requirements, combining with Raman signals originating from the graphene interlayer. Furthermore, owing to the material structure being compatible with MOSFET fabrication, the approach facilitates future integration into integrated circuits, underscoring its significance for practical, hardware‐level, and storage‐free security architectures.

## 4 Materials and Methods

### 4.1 Materials and Laser Irradiation

All Au percolation films were enabled by electron beam deposition with a rate of 0.05 nm s^−1^ on a commercial SiO_2_/Si substrate. The thickness of SiO_2_ layer was 300 nm. Single‐layer graphene was synthesized via chemical vapor deposition. A fs‐laser beam with a pulse duration of 300 fs scans the surface of Au films at a speed of 300 µm s^−1^. The 515‐nm fs‐laser was generated by the second harmonic generation part from a 1030‐nm fundamental laser beam.

### 4.2 Measurement Procedure

A field‐emission SEM (FE‐SEM, Inspect F50) was used to take all SEM images. The reflection spectra were captured through a spectrometer equispped with a reflection probe, designed to detect optical beams that normally reflect off the samples. A 100× objective lens was adopted to obtain the Raman spectra (FEX, NOST) of the SLG by focusing a 531‐nm laser with a power of 0.3 mW.

### 4.3 Calculation and Simulations

The mesh size of 0.5 nm was used in FDTD (Ansys/Lumerical, Canada) simulations for checking the hotspots in Au surfaces. The Python program finished the calculation of hotspots and shortest distances. Another Python program charged the generating a 128‐bit key from optical and electrical PUFs. The simulation and debugging of the Verilog code of AES encryption module were done by the free version of Vivado. Then the Verilog code was executed in a FPGA (Xilinx, xczu3cg‐sfvc784‐1‐e).

## Author Contributions

**Sang‐Shin Lee**: supervision, resources, writing – review and editing. **Shiru Jiang**: conceptualization, investigation, methodology, software, data curation, writing – review and editing, writing – original draft. **Hongliang Li**: data curation, supervision. **Shengjie Ma**: software, formal analysis. **Mengyun Hu**: supervision, writing – review and editing. **Heping Zeng**: conceptualization, supervision. **Lei Wang**: supervision, resources, writing – review and editing.

## Conflicts of Interest

The authors declare no conflicts of interest.

## Supporting information




**Supporting File 1**: advs75973‐sup‐0001‐SuppMat.docx.

**Supporting File 2**: advs75973‐sup‐0002‐MovieS1.mp4.

## Data Availability

The data that support the findings of this study are available on request from the corresponding author. The data are not publicly available due to privacy or ethical restrictions.
