# Supplementary material for: Femtosecond‐Laser‐Induced Physical Unclonable Random Maze Structure for Storage‐Free Encryption
Source: Adv Sci (Weinh). 2026 Jun 11;13(49):e75973. doi: 10.1002/advs.75973 (PMC13336482; doi:10.1002/advs.75973)
Supplement: Supplementary file 1 [file ADVS-13-e75973-s001.docx]

Supporting Information

Femtosecond-laser-induced physical unclonable random maze structure for storage-free encryption

Shiru Jiang, Hongliang Li, Shengjie Ma, Sang-Shin Lee, Lei Wang*, Mengyun Hu*, and Heping Zeng*

*Corresponding authors. E-mail: [wangl@qust.edu.cn](mailto:wangl@qust.edu.cn); [myhu@phy.ecnu.edu.cn](mailto:myhu@phy.ecnu.edu.cn); [hpzeng@phy.ecnu.edu.cn](mailto:hpzeng@phy.ecnu.edu.cn)

**Figure. S1.** Statistical feature size of RMS with different thicknesses of Au film. The thicknesses of Au film are **a** 5 nm, **b** 10 nm, and **c** 20 nm, respectively. The size of the inserted SEM images is 4 ⅹ 4 μm^2^.

**Figure. S2.** SEM images of Au near-percolation film after the 515-nm fs-laser treatment with different pulse energies. The thickness of Au near-percolation film is 20 nm, and NA of the objective lens is 0.6.

**Figure. S3.** SEM images of Au near-percolation film after the 515-nm fs-laser treatment with different objective lenses in NA values. The thickness of Au near-percolation film is 20 nm.

**Note 1:** **Analysis of electron and lattice temperatures for nanostructured and bulk Au**

As is well known, different thermodynamic subsystems dominated by electrons and lattices should be analyzed when it comes to the irradiation originating from a femtosecond laser (fs-laser). The two-temperature model (TTM) is an authoritative tool for figuring out evolutions of spatiotemporal electron temperature (*T_e_*) and lattice temperature (*T_l_*) [1]. Thus, heat transfer processes sponsored in nanostructured and bulk Au layers are demonstrated by solving the following series of equations:

$$C_{e}\frac{{\partial T}_{e}}{\partial t}=\nabla\left( K_{e}{\nabla T}_{e} \right)-g\left( T_{e}-T_{l} \right)+Q\left( t, x \right)$$

(1)

$$C_{l}\frac{{\partial T}_{l}}{\partial t}=g\left( T_{e}-T_{l} \right)$$

(2)

Where *C* and *K* represent the specific heat capacity and thermal conductivity of the gold, respectively. Subscripts *e* and *l* denote the electron and lattice, respectively, and *g* stands for the electron-phonon coupling constant. In addition, $Q\left( t,x \right)$ is the laser source term and defined for a Gaussian beam in time and space:

|  | $Q\left( t,x \right)=\frac{\left( 1-R \right)F}{\tau_{p}\delta_{p}}\exp\left[ -4\ln2\left( \frac{t}{\tau_{p}} \right)^{2}-\frac{x}{\delta_{p}} \right]$***·***$S(x)$ | (3) |
| --- | --- | --- |

Where *R* and *δ_p_* are the optical reflectance and penetration depth of Au near-percolation film with a thickness of 20 nm, respectively, irradiated by a plane wave with a wavelength of 515 nm. *F* and *τ_p_* are the fluence and pulse duration of the used fs-laser, respectively. *t* is the elapsed time after laser irradiation, and *x* is the lateral distance from the site of laser irradiation. $S\left( x \right)$ is utilized to distinguish the influence of the geometric shape of the Au structure on the heat source:

$$S_{bulk}(x)=1$$

(4)

$$S_{nano}=A_{hotspot}exp\left( -\frac{x^{2}}{2\sigma^{2}} \right)$$

(5)

Where$S_{bulk}(x)$ and $S_{nano}$are modulation on laser sources in bulk and nanostructured cases, respectively. *A_hotspot_* = 6 is the enhancement factor due to the hotspot absorption, whose value is confirmed by the maximum to the minimum in the color bar in Fig. 2(b) in the main manuscript. $exp\left( -\frac{x^{2}}{2\sigma^{2}} \right)$ is a Gaussian expression with a Gaussian width of 10 nm, which simulates the physical interception of heat sources due to the geometric shapes of nanostructured Au. All equations were solved using MATLAB, and the related parameters used for the calculations are listed in Table S1. The time step and mesh resolution were set at 10 fs and 2 nm, respectively. Under the irradiation of a 515-nm fs-laser, changes in electron and lattice temperatures in response time and distance for the cases of bulk and nanostructured Au layer are included in Figure S5.

**Table S1.** Parameters for TTM simulations.

| **Parameter** | **Value** | **Unite** | **Reference** |
| --- | --- | --- | --- |
| *C_e_* | 71*T_e_* | J m^–3^ K^–2^ | [2] |
| *C_l_* | 2.45×10 | J m^–3^ K^–2^ | [2] |
| *K_e_* | 317*T_e_/T_l_* | W m^–1–^K^–1^ | [2] |
| *g* | 2.2×10^6^ | W m^–1^K^–1^ | [2] |
| *R* | 0.3 | _ | Measured |
| *δ_p_* | 20 | nm | Measured |
| *τ_p_* | 300 | fs | Measured |
| *F* | 4.5 | J m^-2^ | Measured |

**Figure. S4.** **a** Distribution and density (*ρ*) of hotspots calculated by the algorithm combining adaptive threshold and local maximum, based on the electric (E) field distributions of Au surfaces as shown in the main manuscript Fig. 2(b). **b** Statistical shortest distances between hotspots marked in **a**. The “Ave” represents the average value of the statistical shortest distance.

**Figure. S5.** Calculated electron and lattice temperatures of **a** bulk gold and **b** nano gold as a function of time. Calculated electron and lattice temperatures of **c** bulk gold and **d** nano gold as a function of lateral distance from the focal volume of the fs-laser beam.

**Figure. S6.** SEM images of Au near-percolation film after the 1030-nm fs-laser treatment with different pulse energies. The step of pulse energies is the minimum value in our fs-laser system. The thickness of the Au near-percolation film is 20 nm, and NA of the objective lens is 0.6.

**Figure. S7.** Schematic illustration of the beam overlap ratio, defined as the beam diameter divided by the spatial interval between the *i*^th^ and (*i* + 1)^th^ scanning tracks.

**Figure. S8.** SEM images of Au near-percolation film after the 515-nm fs-laser treatment under **a** single scanning and (**b**-**f**) multiple scanning with different beam overlap ratios. The thickness of Au near-percolation film is 20 nm, and the NA of the objective lens is 0.6.

**Figure. S9.** Schematic of fabricating the random damages on graphene leveraging the optothermoplasmonic effect during the formation of Au RMSs.

**Figure. S10.** Optical micrographs of typical Au RMSs fabricated by LSPR-FLDW under **a** 0.6-NA and **b** 0.3-NA objective lenses. **c** & **d** Measured Raman spectra of single-layer graphene according to numerical indicators in **a** and **b**, respectively. Each Raman spectrum was measured at the center of its corresponding region using the same labels.

**Figure. S11.** The portable microscope that can be mounted on a mobile for conveniently obtaining Au RMS images.

**Note 2: Cost analysis of the low-cost optical/electrical RMS-PUF mode**

In the low-cost optical/electrical mode, the cost was estimated excluding Raman readout, graphene transfer, and encapsulation. The cost was divided into two parts: one-time fabrication cost and repeated operational/readout cost. In this mode, the area of a single RMS is 125×125 μm², and the thickness is 20 nm. The fs-laser was used only for RMS fabrication, with a scanning speed of 300 μm/s and a scanning interval of 1.5 μm, whereas acquisitions of optical and electrical PUFs require portable optical imaging and electrical resistance measurement.

**Table S2**. Fabrication cost of a single RMS

| **Item** | **Basis** | **Estimated cost** |
| --- | --- | --- |
| SiO_2_/Si substrate | 119 RMB per 4-inch wafer | 2.29 × 10^-4^ RMB |
| Au material | 1000 RMB/g | 6.04 × 10^-6^ RMB |
| Au deposition | In-house deposition | Included in overhead |
| Fs-laser writing | 500 RMB/h | 4.8076 RMB |
| Total | - | 4.8079 RMB |

**Table S3**. Operation cost of a single RMS per use

| **Item** | **Basis^a^** | **Estimated cost** |
| --- | --- | --- |
| Smartphone-assisted microscope | 382.13 RMB, 10000 uses | 0.0382 RMB/use |
| Multimeter | 45 RMB, 10000 uses | 0.0045 RMB/use |
| Total | - | 0.0427 RMB/use |

***a*** Assuming both smartphone-assisted microscope and multimeter can be used 10000 times.

**Figure. S12.** Flow chart of generating the optical, electrical, and AES keys.

**Note 3:** **Calculation of** **bit uniformity, inter-HD, and intra-HD**

For a generated binary response $S_{i}=\{s_{i,1},s_{i,2},...,s_{i,L}\}$, where $L$is the sequence length and $s_{i,l}\in\{0,1\}$, Bit uniformity demonstrated in Fig. 3(c) is calculated as:

$$\mathrm{Bit} \mathrm{uniformity}=\frac{1}{L}\sum_{l=1}^{L} S_{i,l}$$

(6)

The inter-HD, was used to evaluate the uniqueness among different PUFs. For two different PUF responses $S_{i}$ and $S_{j}$, the normalized inter-HD was calculated as:

$$HD(S_{i},S_{j})=\frac{1}{L}\sum_{i=1}^{L} (S_{i,l}\bigoplus S_{j,l}), i\neq j$$

(7)

where ⊕ denotes the XOR operation. The average inter-HD over all pairs of *N* independent PUFs was calculated as:

$$inter-HD(S_{i},S_{j})=\frac{2}{N(N-1)}\sum_{i=1}^{N-1} \sum_{j=i=1}^{N} HD(S_{i},S_{j})$$

(8)

The intra-chip Hamming distance, intra-HD, was used to evaluate the reliability of repeated measurements from the same PUF. For the *i^th^* PUF repeatedly measured *M* times, the *r^th^* measured response is denoted as:

$$S_{i}^{(r)}=\left\{ S_{i,1}^{\left( r \right)},S_{i,2}^{\left( r \right)},\ldots,S_{i,L}^{\left( r \right)} \right\}, r=1,2,\ldots M$$

(9)

taking the first measurement *S_i_^(1)^* as the reference response, the intra-HD of the *r^th^* repeated measurement was calculated as:

$${HD}^{r}(S_{i}^{\left( 1 \right)},S_{i}^{(r)})=\frac{1}{L}\sum_{i=1}^{L} (S_{i,l}^{(1)}\bigoplus S_{i,l}^{(r)}), r=2, 3,\ldots,M$$

(10)

the average intra-HD was then calculated as:

$$Intra-HD(S_{i}^{\left( 1 \right)},S_{i}^{(r)})=\frac{1}{M-1}\sum_{r=2}^{M} {HD}^{(r)}, r=2, 3,\ldots,M$$

(11)

**Note 4: Statistical randomness and unpredictability RMS-based PUFs**

To further quantify the information content and statistical randomness of the generated binary responses from RMS-based PUFs in terms of response randomness, worst-case predictability, and statistical non-random patterns were analyzed. For a binary response with a bit uniformity of *p*, where *p* denotes the fraction of “1” bits, the Shannon entropy per bit was calculated as [3]:

$$H=-p\log_{2} p-(1-p)\log_{2} p(1-p)$$

(12)

and the min-entropy per bit was calculated as [4]:

$$H_{min}=-\log_{2} p[max\left( p,1-p \right)]$$

(13)

The Shannon entropy quantifies the average information content of each response bit, whereas the min-entropy provides a conservative estimate of the worst-case predictability. As shown in Figure S13, both Shannon entropy and min-entropy are concentrated near the ideal value of 1, with average values of 0.999175 and 0.960151, respectively. These results indicate that the generated optical-PUF responses exhibit high information content and low worst-case predictability.

In addition, the National Institute of Standards and Technology test suite (NIST SP 800−22) for statistical randomness tests was executed [5,6]. We chose the seven most commonly used items from 15 distinct components comprised by NIST, as shown in Table S2, which examines different types of possible non-random behavior, including global bit imbalance, local block-level bias, cumulative random-walk deviation, abnormal run-length statistics, and nonuniform local bit-pattern distributions. As summarized in Table S2, all tested items passed the NIST-style randomness criteria. Specifically, 90/90 sequences passed the frequency, block frequency, runs, serial, and approximate entropy tests, while 89/90 sequences passed the cumulative sums and longest run tests. Since the passing proportions exceed the minimum acceptable passing rate of 88/90, the generated 256-bit optical-PUF responses can be considered statistically random under the applied NIST-style test conditions.

**Figure. S13.** **a** Shannon entropy and **b** Min-entropy extracted from 90 optical PUF images.

**Table S4**. NIST SP 800-22 statistical test of binary sequences generated from optical-PUFs

| **NIST Statistical Test*^a^*** | **M or m** | ***p*-Value** | **Proportion** | **Result*^b^*** |
| --- | --- | --- | --- | --- |
| Frequency | - | 0.669655 | 90/90 | Pass |
| Block frequency | 16 | 0.493641 | 90/90 | Pass |
| Cumulative sums | - | 0.245834 | 89/90 | Pass |
| Runs | - | 0.384849 | 90/90 | Pass |
| Longest runs | 8 | 0.263581 | 89/90 | Pass |
| Serial | 2 | 0.599603 | 90/90 | Pass |
| Approximate entropy | 2 | 0.506925 | 90/90 | Pass |

***a*** 90 independent 256-bit binary sequences extracted from 90 different optical-PUFs were evaluated. The response was considered to pass an individual test when the corresponding *p*-value ≥0.0001. ***b*** The overall test was considered passed when the passing proportion exceeded the minimum acceptable passing rate of 88/90.

**Note 5:** **Long-term stability and environmental robustness of** **RMS-based PUFs.**

To evaluate the long-term stability and environmental robustness of the RMS-based PUFs, the normalized Lempel–Ziv (LZ) entropy and bit error rate (BER) were calculated for the optical and electrical PUF-derived keys over time and under different temperatures. The normalized LZ entropy was used to quantify the complexity of the generated binary sequence. Based on the LZ parsing complexity of finite sequences, the normalized LZ entropy was calculated as [7,8]:

$$H_{LZ}=\frac{C_{LZ}(S)\log_{2} L}{L}$$

(14)

where *S* is the generated binary sequence, *L* is the sequence length, and *C_LZ_*(S) is the Lempel–Ziv parsing complexity of *S*. A value of *H_LZ_* close to 1 indicates high sequence complexity and randomness. The BER was used to evaluate the reproducibility of the regenerated key and was calculated as [9,10]:

$$BER=\frac{1}{L}\sum_{i=1}^{L} K_{i}^{(0)}\bigoplus K_{i}^{(t)}$$

(15)

where *K^(0)^* is the reference key, *K^(t)^* is the regenerated key measured after a given time or under a given temperature, and ⊕ denotes the XOR operation. An ideal BER value of stable PUFs should be 0.

As shown in Figs. S14(a) and (c), both optical and electrical PUF-derived keys maintained stable LZ entropy and low BER over 210 days at room temperature. The average LZ entropy values were 0.8975 and 0.87725 for the optical and electrical PUFs, respectively. Meanwhile, the average BER values were 0.0905 for the optical PUF and 0.05625 for the electrical PUF, indicating reliable key regeneration over long-term storage. Similar stability was observed under temperature variations from 0 to 55 °C after both 1 day and 210 days. The average LZ entropy values remained close to 0.895 for the optical PUF and 0.879 for the electrical PUF, while the corresponding BER values remained low at approximately 0.089 and 0.057, respectively. These results confirm that the RMS-based PUFs preserve their sequence complexity and key reproducibility under temporal drift and thermal perturbations, demonstrating excellent long-term stability and environmental robustness. To mitigate possible effects from surface contamination, moisture, and mechanical abrasion, appropriate encapsulation or protective packaging may be introduced in future practical applications. Similar protective strategies have been explored in Au-based nanostructures, among which the deposition of a thin or ultrathin silica layer on Au surfaces represents a simple and effective approach to improve structural and environmental stability[11].

**Figure. S14.** **a** Lempel-Ziv (LZ) entropy and **c** bit error rate of electrical and optical over 210 days under room temperature (25 ℃). **b** LZ entropy and **d** bit error rate of electrical and optical for the temperature range from 0~55 ℃ after 1 and 210 days.

**Note 6:** **On-site generated PUF key in** **storage-free** **asymmetric encryption for information communication**

Modern cryptographic systems are generally classified into symmetric and asymmetric encryption schemes, depending on whether the same key is used for both encryption and decryption. In an asymmetric scheme, encryption and decryption are enabled by the public and private keys, respectively[12]. The public key is mathematically derived from the private key through a one-way hard problem, such as the elliptic curve digital signature algorithm (ECDSA) [13], which allows it to be openly transmitted without compromising security. But the private key must be securely protected. However, with the advent of quantum computing and the increasing computational power available to adversaries, stored private keys have become increasingly susceptible to exposure.

As illustrated in Fig. S15, two communicators obtain their respective optical and electrical PUFs from distinct RMS_1 and RMS_2. After binarization, hashing, and scrambling, each side generates its own private keys, from which the corresponding public keys are derived via elliptic curve operations and exchanged. Then both parties use the other’s public key for encryption and their own private key for decryption, enabling secure information exchange. After communication, all keys are erased to eliminate long-term storage risks and are regenerated for the next communication.

**Figure. S15.** Diagram for information communication in a storage-free asymmetric encryption using on-site generated key based on Au RMS PUFs.

**Table S5**. Signals of AES/FPGA encryption control module.

| **Signal Name** | **Transmission Direction** | **Signal Meaning** |
| --- | --- | --- |
| clk | input | Clock signal |
| rst | input | Reset signal |
| load | input | Data load enable signal, used to control input plaintext/ciphertext, key, S-box configuration data, etc |
| address | input | Registry or RAM address, used to represent plaintext/ciphertext/key register, S-box RAM cell |
| keyexp | input | Key expansion enables signal |
| staenc | input | Start encryption enable signal |
| stadec | input | Start decryption enable signal |
| keyexprdy | output | Key expansion complete flag signal |
| encdecrdy | output | Encryption/decryption operation complete flag signal |
| keysel | output | Round key selection signal, keysel=0 selects the seed key in the key register; otherwise, it selects the round key after key expansion transformation. |
| **rndkren** | output | round key register write enable signal |

**Figure. S16.** Circuit schematic of AES encryption/decryption module.

**Table S6**. External signals of AES encryption/decryption module.

| **Signal Name** | **Transmission Direction** | **Signal Meaning** |
| --- | --- | --- |
| wrsben | input | Enable signal for writing configuration data to S-box |
| wrsbaddr | input | Address of S-box configuration data |
| sbdata | input | S-box configuration initial data |
| keyadsel | input | Selection signal for input data in key addition operation |
| mixsel | input | Choose whether to perform an inverse column hybrid transformation (decryption process) |
| reginsel | input | Selection signal for the input data of the result register |
| wrregen | input | Result register write enable signal |
| intxt | input | External input data (plaintext or ciphertext) |
| roundkey | input | Subkey |
| shift | input | Result register shift enable signal |
| outtxt | output | Output data (encryption/decryption results) |

**REFERENCES**

[1] Roberts, A. S. et al. Laser writing of bright colors on near-percolation plasmonic reflector arrays. *ACS Nano* **13**, 71–77 (2019).
[2] Block, A. et al. Tracking ultrafast hot-electron diffusion in space and time by ultrafast thermomodulation microscopy. *Sci. Adv.* **5**, eaav8965 (2019).
[3] Shannon, C. E. A mathematical theory of communication. *Bell System Technical Journal* **27**, 379–423 (1948).
[4] Rényi, A. On measures of entropy and information. *Proceedings of the Fourth Berkeley Symposium on Mathematical Statistics and Probability* **1**, 547–561 (1961).
[5] Kwak, J.-K.et al. All-optical reconfigurable physical unclonable function for sustainable security. *Adv. Mater*. Preprint, 202521712 (2026).
[6] Kim, M. S., et al. Revisiting silk: a lens-free optical physical unclonable function. *Nat. Commun*. **13** (1), 247 (2022).
[7] Lempel, A. and Ziv, J. On the complexity of finite sequences. *IEEE Transactions on Information Theory* **22**, 75–81 (1976).
[8] Kaspar, F., Schuster, H. G. Easily calculable measure for the complexity of spatiotemporal patterns. *Physical Review A* **36**, 842–848 (1987).
[9] Herder, C., Yu, M.-D., Koushanfar, F., Devadas, S. Physical unclonable functions and applications: a tutorial. *Proceedings of the IEEE* **102**, 1126–1141 (2014).
[10] J. Ahn et al., Nanoseed-based physically unclonable function for on-demand encryption. *Science Advances* **11**, eadt7527 (2025).
[11] Ooms, M. D., Jeyaram, Y., Sinton, D. Disposable Plasmonics: Rapid and Inexpensive Large Area Patterning of Plasmonic Structures with CO_2_ Laser Annealing. *Langmuir* **31**(18), 5252–5258 (2015).
[12] Simmons, G.J. Symmetric and asymmetric encryption. A*CM Comput. Surv. (CSUR)* **11**, 305–330 (1979).
[13] Yassein, M. B., Aljawarneh, S., Qawasmeh, E., Mardini, W., Khamayseh, Y. Comprehensive study of symmetric key and asymmetric key encryption algorithms, *2017 International Conference on Engineering and Technology (ICET)*, Antalya, Turkey, 1-7, (2017).
